# Shooting yourself in the foot: How immune cells induce antibiotic tolerance in microbial pathogens

**DOI:** 10.1371/journal.ppat.1009660

**Published:** 2021-07-22

**Authors:** Jenna E. Beam, Sarah E. Rowe, Brian P. Conlon

**Affiliations:** Department of Microbiology and Immunology, University of North Carolina Chapel Hill, Chapel Hill, North Carolina, United States of America; Duke University School of Medicine, UNITED STATES

## Abstract

Antibiotic treatment failure of infection is common and frequently occurs in the absence of genetically encoded antibiotic resistance mechanisms. In such scenarios, the ability of bacteria to enter a phenotypic state that renders them tolerant to the killing activity of multiple antibiotic classes is thought to contribute to antibiotic failure. Phagocytic cells, which specialize in engulfing and destroying invading pathogens, may paradoxically contribute to antibiotic tolerance and treatment failure. Macrophages act as reservoirs for some pathogens and impede penetration of certain classes of antibiotics. In addition, increasing evidence suggests that subpopulations of bacteria can survive inside these cells and are coerced into an antibiotic-tolerant state by host cell activity. Uncovering the mechanisms that drive immune-mediated antibiotic tolerance may present novel strategies to improving antibiotic therapy.

## Why do antibiotics frequently fail to clear infection?

Antibiotic resistance, defined as the genetically heritable capacity to grow in the presence of an antibiotic, is continuing to evolve and spread and represents a major threat to global health [1]. However, high rates of treatment failure are often attributed to antibiotic-tolerant cells, rather than resistance [2,3]. Antibiotic tolerance is the ability of bacterial cells to survive for extended periods in the presence of bactericidal antibiotics [4]. Antibiotic failure occurs in approximately 1 in 5 patients with *Staphylococcus aureus* bloodstream infections, contributing to more than 20,000 deaths annually [2]. Additionally, many bacterial infections respond to antibiotic therapy only for relapse of infection to occur once treatment is ceased [3,5]. While no singular mechanism underlying antibiotic tolerance has been established, evidence strongly suggests that interactions with innate immune cells are major contributors to the phenomenon in vivo [5–9]. Importantly, recent studies also demonstrate the emergence of antibiotic resistance from antibiotic-tolerant reservoirs [10].

Identifying the cause of antibiotic failure in patients relies on further probing interactions between the pathogen, host, and antibiotic. Antimicrobial chemotherapy and bacterial pathogenicity have generally remained separate areas of study that has limited our understanding of how our antibacterials are working, or not working, in the context of the host immune environment.

## How well do antibiotics kill in vivo?

**Fig 1:**
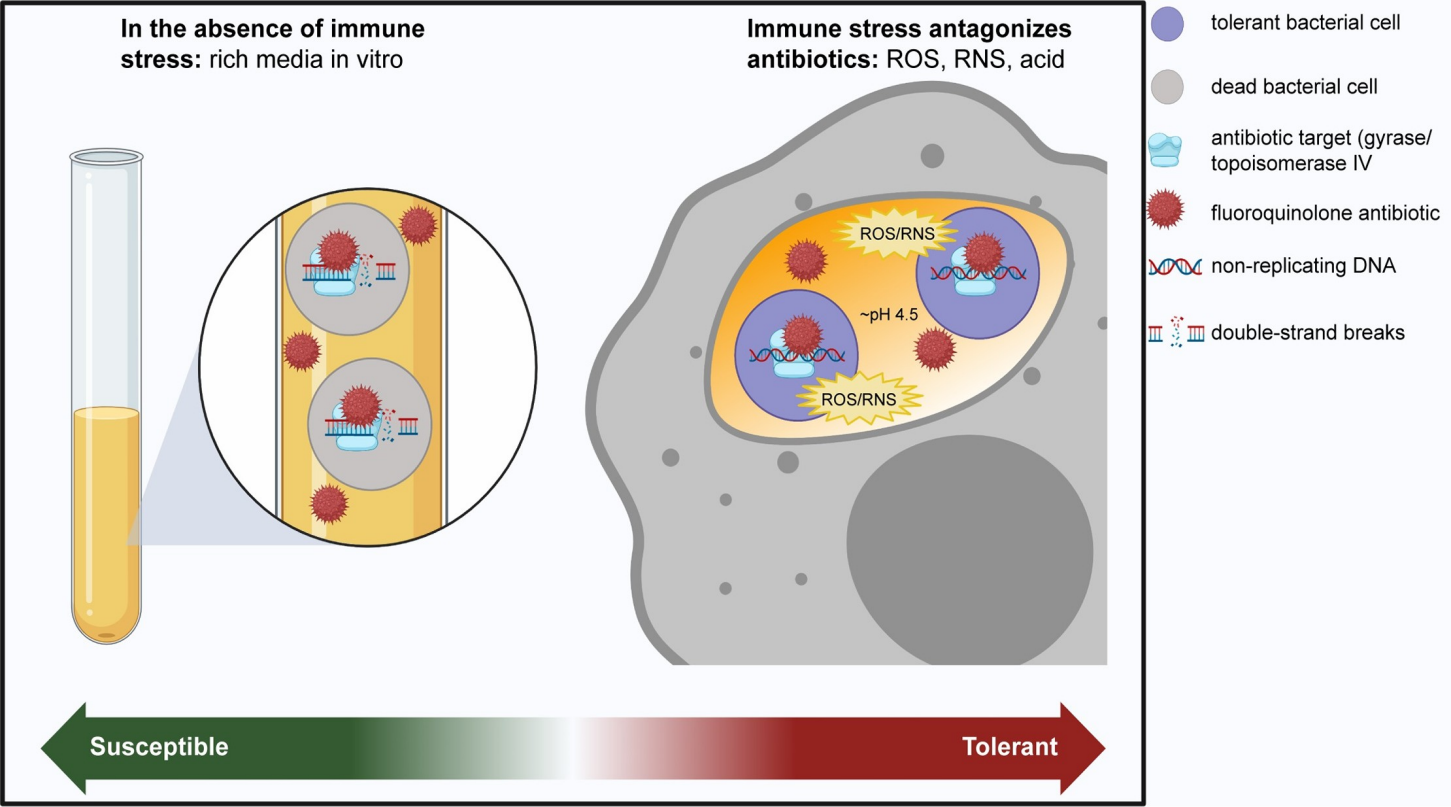
Bactericidal drugs may be static in vivo. In the absence of stress, when bacterial cells are undergoing replication, the bactericidal antibiotic fluoroquinolone binds to its target (DNA gyrase and topoisomerase IV) and prevents the religation step during DNA synthesis. This leads to double-strand breaks and cell death. Macrophage-produced stresses (such as ROS/RNS or acid stress) may down-regulate cellular processes targeted by antibiotics. In the absence of replication, the fluoroquinolone may bind to its target but does not cause double-strand breaks or cell death. This leads to antibiotic tolerance. Figure created using BioRender. ROS/RNS, reactive oxygen and nitrogen species.

Antibiotics have been used to treat patients since the 1940s [11], but how antibiotics kill bacteria in vivo and facilitate infection clearance is still not fully understood. Antibiotics are frequently described as bacteriostatic or bactericidal [12]. Bacteriostatic antibiotics inhibit bacterial growth without causing cell death and hence rely on the immune system to eliminate the infection. Bactericidal antibiotics can directly induce bacterial cell death and most work by corrupting active processes: β-lactams causing a futile cycle of peptidoglycan synthesis and autolysis [13]; aminoglycosides cause mistranslation, resulting in toxic peptides [14]; and fluoroquinolones inhibit the religation step of DNA replication, causing double-strand breaks [15]. A drug is deemed bactericidal if it kills more than 99.9% of an exponential phase population of bacteria during overnight incubation [12]. In that sense, the designation is somewhat arbitrary and is established under in vitro conditions that bear little resemblance to the in vivo host environment. Stresses that slow or stop bacterial processes such as protein synthesis can limit the damage caused by a bactericidal drug, resulting in antibiotic tolerance and effectively reducing bactericidal drugs into static drugs (Fig 1) [16,17]. We find that “bactericidal” antibiotics, such as vancomycin and rifampicin, frequently fail to reduce the bacterial load in mouse models of infection [6,18]. Additionally, many studies report equivalent efficacy of bacteriostatic and bactericidal drugs in patients [12,19,20], which further suggests that bactericidal drugs often may not be “cidal” in vivo. The factors in the infection environment that inhibit bactericidal activity remain poorly understood. Bactericidal activity of antibiotics in vitro at low, physiologically achievable concentrations can rapidly kill bacteria in culture, and, if this cidality were realized in vivo, it could have a major impact on antibiotic treatment duration and efficacy.

## Is the immune response responsible for poor antibiotic efficacy?

Immune cells evoke a plethora of stresses (nutritional immunity, reactive oxygen and nitrogen stress, acid stress, antimicrobial peptides, and proteases) to eliminate invading pathogens, but there is mounting evidence that components of the innate immune response are antagonistic to antibiotics [5–7,9]. It’s been shown that bacterial populations that survive within immune cells are enriched for antibiotic tolerance [6,9]. Multiple pathways to tolerance appear to exist, and the relevance of these pathways vary by pathogen. Macrophages induce antibiotic tolerance of internalized *S*. *aureus* through reactive oxygen species (ROS) that cause collapse of the tricarboxylic acid (TCA) cycle an entrance to a low ATP state [6]. Additionally, activation of the stringent response has also been shown to contribute to *S*. *aureus* intracellular tolerance [7], while neutrophil interaction as well as acid stress have also been shown to induce antibiotic tolerance in *S*. *aureus* abscess patient isolates [21]. In *Salmonella* Typhimurium, antibiotic-tolerant persister subpopulations are induced intracellularly through acid stress, nutritional deprivation, and the activation of toxin–antitoxin modules [9]. Studies in *Mycobacterium tuberculosis* (*Mtb*) suggest that antibiotic tolerance is predominantly mediated through nitrosative stress and is increased following cytokine activation of macrophages or immunization of mice [5].

## How can we improve antibiotic efficacy in vivo?

Identifying the stresses encountered by bacteria, as well as the bacterial response to these stresses, during infection that prevent lethality of antibiotics may be key to improving their therapeutic potential during infection [22]. Several studies, by us and others, have shown that antibiotic efficacy against *S*. *aureus* is improved by reducing phagocytic burst: *S*. *aureus* was more susceptible to antibiotics in *Ncf1*^*−/−*^ and *Nox2*^*−/y*^ mice deficient in oxidative burst [6,23] and within polymorphonuclear leukocytes (PMNs) isolated from patients with chronic granulomatous disease (CGD) [24]. In addition, *Mtb* was more susceptible to antibiotics in macrophages lacking an inducible nitric oxide synthase (*Nos2*^*−/−*^) gene [23]. These studies are crucial to determining the mechanism of antibiotic tolerance during infection and may point toward intervention strategies to improve antibiotic efficacy.

Studies that employ strategies such as treatment with antioxidants [6,25–27] and immunomodulation [28,29] to improve antibiotic efficacy against a variety of pathogens suggest that combining antibiotic treatment with host-targeted therapies has promising therapeutic potential. Immunomodulatory strategies, both stimulation and repression of the innate immune response, have been shown to potentiate antibiotic killing of different pathogens. PPARy agonists that lead to M2-like skewing of macrophages improve immune-mediated clearance of *S*. *aureus* [30]. As decreased ROS production is associated with M2-like macrophages [31] and ROS induce antibiotic tolerance in *S*. *aureus* [6], antibiotic treatment in combination with M2-skewing compounds may represent a viable therapeutic strategy to both improve immune-mediated clearance of *S*. *aureus*, while also increasing antibiotic susceptibility. Another study found that combinatorial treatment with the glucocorticoid dexamethasone and antibiotics led to improved outcome and decreased infection severity in a murine model of *S*. *aureus* arthritis by decreasing macrophage recruitment and inflammation [28]. Glucocorticoids are among the most commonly used anti-inflammatory therapies in medicine with largely inhibitory effects on the immune system [28,32,33]. We also recently showed that Tempol, a superoxide dismutase mimetic and potent antioxidant, improved antibiotic efficacy in a systemic *S*. *aureus* infection [6]. However, differences in host genetics may profoundly affect the success of immunomodulatory strategies [34].

Similar to *S*. *aureus*, host-derived reactive species have been shown to induce the formation of *Mtb* persisters [5]. Additionally, high levels of oxidative stress are commonly found in patients with tuberculosis (TB) [35]. The use of small molecule thiols, such as N-acetylcysteine (NAC), has been shown to increase clearance of *Mtb* in the absence of antibiotics while also preventing the formation of *Mtb* persisters [25–27]. In addition, natural killer (NK) cells treated with NAC up-regulated the production of cytotoxic ligands that prevented growth of *Mtb* in human monocytes [27]. NAC also reduces the production of reactive species by the host [26] and improves antibiotic efficacy against *Mtb* [25], suggesting that it may broadly improve antibiotic efficacy against other pathogens that exhibit tolerance following ROS exposure.

Although the reduction of ROS appears to be advantageous for the clearance of *Mtb* and *S*. *aureus* infection, this may not hold true for all pathogens. *S*. Typhimurium persisters reprogram the macrophage response from a pro-inflammatory to an anti-inflammatory state, dampening the antimicrobial strategies of the macrophages and allowing slow-growing *Salmonella* persisters to evade both antibiotic and immune-mediated killing [8]. As *Salmonella* persisters are able to survive by shifting the macrophage response away from a pro-inflammatory state, it reasons that restimulation of a pro-inflammatory immune response may improve killing of *S*. Typhimurium persisters.

Although a lot remains to be learned, targeting the host response to bacterial infection will likely increase the efficacy of existing antibiotics, an intriguing strategy given the shortage of new and effective antibiotics in development [36].

## Discussion

Interactions between the innate immune system and bacterial pathogens have definite impacts on antibiotic efficacy. This realization opens the door to using immunomodulators to maximize antibiotic efficacy to improve the treatment of infection. Ideally, a specific immunomodulator would increase antibiotic susceptibility of a specific pathogen without any negative impacts on the hosts’ ability to fight the infection. If antibacterial strategies associated with activated immune cells are driving tolerance, is acute immunosuppressive therapy in combination with bactericidal antibiotics a viable treatment option for *S*. *aureus* and *Mtb*? Or in the case of *Salmonella*, is amplification of the pro-inflammatory response a better treatment strategy?

The potential of immunomodulatory strategies to improve antibiotic efficacy is appealing, but immunomodulation during bacterial infection is certainly complicated and not without risk. Although there is more work to be done to understand potential challenges and drawbacks of immunomodulation, this strategy has been a game changer for patients living with other diseases such as rheumatoid arthritis, psoriasis, ulcerative colitis, Crohn disease, and various types of cancers [37–39]. HUMIRA, developed by AbbVie, blocks tumor necrosis factor alpha (TNF-α) and reduces inflammation associated with many autoimmune disorders. Despite the increased risk of respiratory infections and some cancers, HUMIRA remains the top-selling drug in the United States due to its ability to elevate patients’ quality of life [40]. Additionally, increased understanding of the tumor microenvironment has led to the coupling of immunomodulatory therapies with chemotherapy (“chemoimmunotherapy”) for the treatment of different cancers [41–43]. For example, squamous cell lung carcinoma represents up to 30% of all non-small cell lung cancers, yet treatment options are limited and mostly ineffective [44]. Squamous cell lung carcinoma tumors are more resistant to immunotherapy, and traditional chemotherapy treatments administered at the maximum tolerated dose are highly toxic to the patient with little effect on the tumor [44]. However, recent clinical trials have shown that coupling traditional chemotherapy with immunomodulatory therapy significantly increased patient survival [44]. Following the preclinical and clinical success of immunomodulation therapies for other diseases, it is possible that immunomodulation may be the breakthrough strategy for unleashing the lethality of antibiotics.
